#  Cytogenetic Abnormalities with Interphase FISH Method and Clinical Manifestation in Chronic Lymphocytic Leukemia Patients in North-East of Iran

**Published:** 2017-07-01

**Authors:** Hossein Rahimi, Mohammad Hadi Sadeghian, Mohammad Reza Keramati, Amir Hossein Jafarian, Sepideh Shakeri, Seyyede Fatemeh Shams, Neda Motamedi, Maryam Sheikhi, Hossein Ayatollahi

**Affiliations:** 1 MD, Associate Professor of Internal Medicine, Faculty of Medicine, Mashhad University of Medical Sciences, Mashhad, Iran; 2 Msc, Cancer Molecular Pathology Research Center, Department of Hematology and Blood Bank, Faculty of Medicine, Mashhad University of Medical Sciences, Mashhad, Iran; 3 MD, Associate Professor of Hematopathology, Cancer Molecular Pathology Research Center, Department of Hematology and Blood Bank, Faculty of Medicine, Mashhad University of Medical Sciences, Mashhad, Iran

**Keywords:** Chromosomal aberration, chronic lymphocytic leukemia (CLL), Interphase FISH (I-FISH), Polymerase chain reaction (PCR)

## Abstract

**Background: **Chronic lymphocytic leukemia (CLL) is one of the most prevalent adult leukemias. This malignancy is known by lymphocytosis for a duration of more than 3 months. In fact, it is a heterogeneous clinical disease with changeable progression. Chromosomal aberrations are significant parameters to predict result and survival rate and find treatment strategies for each patient. Cytogenetic methods are known as sensitive and relatively new procedures to detect abnormalities in genome.

**Materials and Methods:** In order to identify CLL-related chromosomal abnormalities, 48 CLL patients included 38 Men and 10 Women with mean age of 58.25±36 were enrolled in this case series study.The survey was done at Cancer Molecular Pathology Research Center, Mashhad University of Medical Sciences. Interphase fluorescent in situ hybridization (I-FISH) was done on unstimulated peripheral blood or bone marrow samples, which were cultured in whole medium culture; it was used to detect chromosomal abnormalities such as 11q- , 13q14-, 17p- , 6q- and trisomy 12 in CLL patients.

**Results:** Analysis demonstrated that 45.5% of CLL cases had chromosomal abnormalities; 13.63% haddel 17p, 40.90% had del 13q14 and 9.09% had del 11q. Statistical analysis of data revealed a significant relevancy between age variable and splenomegaly occurrence (P value<0.05). The younger the patients were, the less the splenomegaly occurrence.

**Conclusion: **Laboratory findings were correlated with clinical data.

## Introduction

 Chronic lymphocytic leukemia (CLL), which includes more than 30% of adult leukemias, is one of the most prevalent adult leukemias in western world^1^^-^^4^.The disease is highly variable from indolent patients to cases with aggressive and progressing stages of disease; this heterogeneity makes a significant influence on treatment strategies, clinical approaches and overall survival time from disease diagnosis. Acquired chromosomal aberrations play a significant role in CLL pathogenesis. CLL is represented by a comparative stable genome in contrast with other malignancies or solid tumors^5^.

This malignancy is recognized in patients (usually over 50 years old) by lymphocytosis (30×10^9 ^per liter) for a duration of more than 3 months^6^. Among all known clinical features; lymphadenopathy, organomegaly, fever and fatigue have been detected as the most prevalent symptoms in CLL^4^ though 25% of patients are asymptomatic^6^.

In order to anticipate survival rate of CLL patients, clinical staging system designed by Rie et al. and Binet et al. is used^4^^,^^6^^-^^10^,however, it is a heterogeneous clinical disease with variable progression from months to more than 10 years ^6^^, ^^9^^, ^^10^ . Some reports suggest that disease staging and chromosomal aberrations are absolutely relevant^11^.

 In general, factors such as CD38, mutant region of immunoglobulin heavy chain gene (IGHV), ZAP-70, chromosomal aberrations and complex karyotype, are considered as prognostic markers ^9^^, ^^12^^, ^^13^^,^^15^^, ^^16^^, ^^17^.

Chromosomal changes have been reported in 40-50% of CLL patients^6^^,^^9^. The most recurrent chromosomal abnormalities are partial loss of one chromosome, such as deletions on 6q, 11q, 17p and 13q, gains of whole chromosome such as trisomy 12^18^. One particular study mentioned that specific cytogenetic abnormalities like 11q or 17p deletions are presented with poor clinical outcome, moreover, mutations in certain genes such as TP53 are responsible for poor prognosis. New studies which use next generation sequencing (NGS) technique have identified new gene aberrations such as NOTCH1 and SF3B1 mutations like BIRC3 disruptions that may lead to some clinical heterogeneities^5^.

Del 6q is seen in lymphoid malignancies such as multiple myeloma (MM),^2^waldensterommacroglobolinemy (MW), acute lymphocytic leukemia (ALL), and mantle zone lymphoma (MZL) which can be helpful in malignancies prognosis. Chromosomal aberrations are significant parameters to predict result, survival rate, and find treatment strategies for each patient^1^^,^^3^.

 More than 30 years ago before propagation of molecular methods, no chromosomal changes were assumed, but nowadays their prognostic roles are approved^19^. In 1990, Tuliusson et al used conventional cytogenetic techniques to determine the role of chromosomal abnormalities in the prognosis of malignancies. More than 50% of CLL patients had clonal chromosomal abnormalities in this karyotype banding analysis^5^.

In 2000, Dohner et al. introduced Interphase fluorescent in Situ Hybridization (I-FISH) technique as a method to detect chromosomal abnormalities. This technique detected chromosomal disorders in 80% of patients^1^. Recently, I- FISH has been developed which can be applied to interphase cells; I-FISH does not need any stimulations in culture process and can be performed on the unstimulated cells. Due to weak stimulation of B lymphocytes and low mitotic index in conventional cytogenetic studies, it is known as high sensitive method for evaluation of B cells cytogenetic abnormalities^12^. Further advantages of this method have been mentioned in the discussion part of current manuscript.

 The aim of this study was to detect cytogenetic abnormalities of CLL patients by I- FISH method, and determine the relevance of clinical signs and genomic types of abnormalities in North-East of Iran.

## MATERIALS AND METHODS

 This case series study was conducted at Cancer Molecular Pathology Research Center, Mashhad University of Medical Sciences during 2014-2016. All studied individuals were new cases and had not taken any treatment. Forty-eight CLL patients including 38 men and 10 women with mean age of 58.7±5 and 64.9±1, respectively were enrolled in this case - series study. CLL diagnosis was done by two expert hematopathologists based on the following criteria: lymphocytosis of 5× 10^9^ /L in peripheral blood or bone marrow, and positive markers of CD5, CD19, CD20 and CD23 as the results of immunophenotype study.

**Conventional cytogenetic**

Peripheral blood or bone marrow heparinized specimens were cultured in 5 ml completeculture medium consisting of RPMI-1640 medium (Gibco-USA) supplemented with 10% fetal bovine serum (Gibco, USA), 1% penesterep antibiotic (Gibco, USA) and 1% L-glutamine (Gibco, USA) . The cultured samples were incubated in 37°C for 24 hours without any mitogens.

Harvesting and slide preprartion were done according to the standard cytogenetic methods (Hypotonic treatment and methanol-acetic acid, Merk Germany; 3:1 ratio of fixation) (0.075 M KCL). Treatment and fixation were done using Carnoy's fixative. Six slides were prepared for each cultured sample. FISH analysis was done for all slides.

**Interphase fluorescent in situ hybridization**

To indicate del 17p13, del 13q14, del 11q22, del 6q21 and trisomy12, dual-color probes and their control probes were purchased from Kreatech Company and were used according to the manufacturer’s instructions. The control probs were as follows: Chromosome 12 centromere for trisomy 12, 13q14 (DLEU) for 13q34 and reverse, centromere 17 for 17p13 deletion (p53) centromere11 for 11q22 (ATM) and centromere 6 for 6q21. Slides and probes were co-denatured at 77°C with thermo-rite (Kreatech, Amesterdam, and Netherland) and hybridized at 37°C for 16 hours. A total of 200 nucleuses were counted to determine cut-off values for FISH analysis according to the Smoley's research. The upper limits of normal cut off were set at 6% for 6q- , 2%, 0.5, 2 and 5% for 11q- , +12, 13q14 and 17p- , respectively^20^. Olympus BX41 florescence microscope (Japan) and video test software (version 3.1) were used for imaging procedure and the results were reported according to ISCN 2013. 

**Statistical analysis**

The data were analyzed using SPSS package (version 14.9; spss, Chicago, IL).P values less than 0.05 were considered statistically significant.

## Results

 Forty-eight samples obtained from patients were analyzed using cytogenetic culture and FISH technique. Sex ratio was 4.5 (38 males and 10 females). Patients were 45 to 71 years old with mean age of 58.7±5 and 64.9±1 in male and female groups, respectively. Laboratory findings are cited in Table 1.

**Table1:** laboratory finding of CCL patient in north east of Iran comparison to CI

**Patients**	**CI**	**Variable**
M:10.93 F:11.00	M: 11.50-15.80 F: 11.50-15.30	**Hb (gr/dl)**
160	4.40-11.30	**WBC (10** ^3^ **/mm3)**
182	172 -450	**PLT(10** ^3^ **/mm3)**

M: Male, F: Female, CI: Confidence interval

**Fig1:**
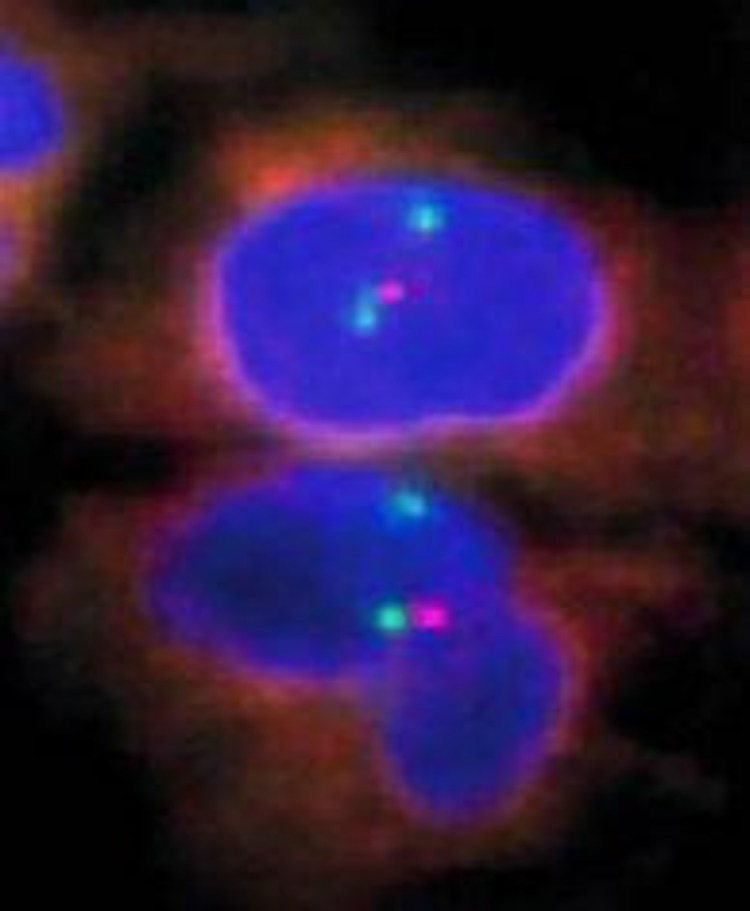
del 13q14,nucish(DLEU×1,13q34×2)[100]

**Fig2:**
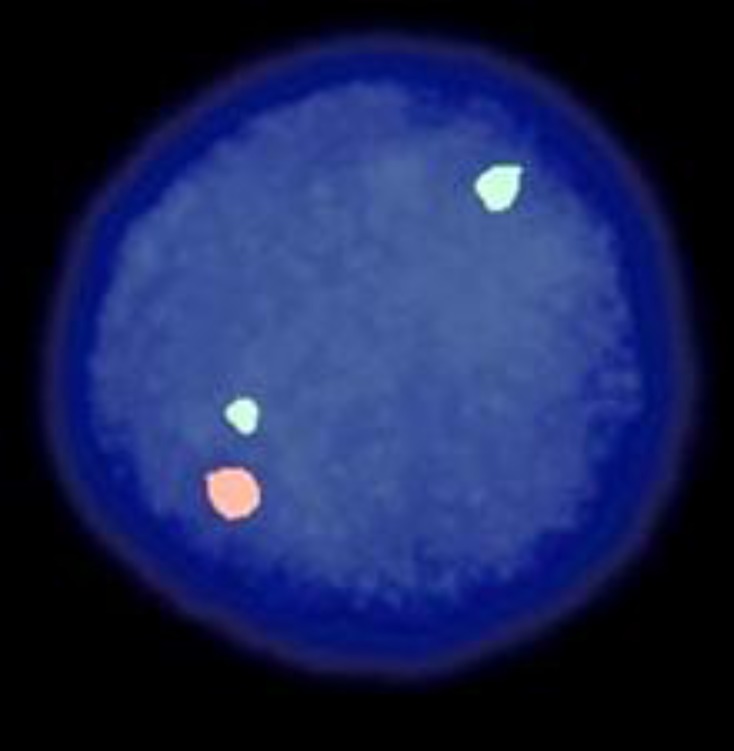
del11q22,nucish (ATM×1,SE11×2)[100]

**Fig3:**
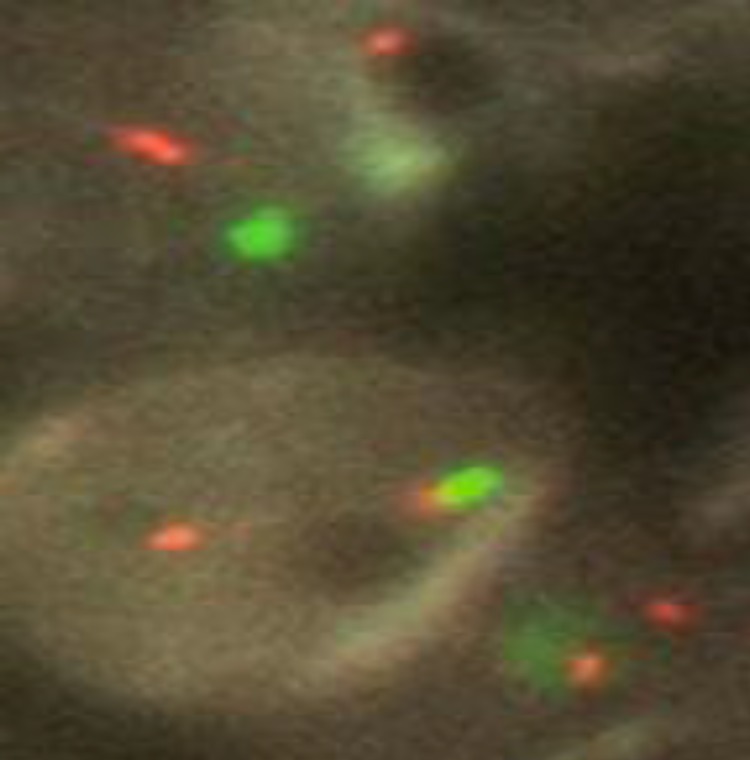
del 17p13, nucish(P53×1,SE17×2)[100]

I- FISH was done for del 17p, del 13q14, del11q, del 6q and trisomy 12 on 24-hour unstimulated culture. Analysis demonstrated that 20 out of 48 male patients do not have any chromosomal aberrations. 13.63% of cases had del 17p, 40.90% had del 13q14 and 9.09% had del 11q (Figures 1, 2 and 3). Totally, chromosomal aberrations were observed in 54.54% of studied population. No del6q21 and trisomy 12 were detected in the studied cases. The most common reported rearrangement was del13q14 which was found in 31.56% of men and 60.00% of women. 

37.50% of lymphadenopathies were seen in more than one site. Cervical lymphadenopathy was the commonest type among patients. Spleen assessment demonstrated splenomegaly in 54.54% of cases. The youngest patient, 45 years old, had no lymphadenopathy and splenomegaly; no chromosomal changes were seen in this case. WBC count was the highest in del 13q14, but the lowest count was seen in del11q. The highest hemoglobin concentration was seen in cases with del 17p, while anemia was more obvious in cases with del13q14. It is necessary to note that only one patient who was a 65-year-old female had del13q14 and del17p abnormalities together.

It is evident that not only confidence interval for hemoglobin concentration is broader in men but also the reduction is more common in men. More clinical and laboratory information are presented in Table 2. 

**Table 2:** Clinical, laboratory information of patients

**NOS**	**11q**	**13q**	**17p**	**variable**
20	4	18	6	Number
53.50	63.20	65.33	63.00	Median age
20	4	12	2	Male
0	0	6	4	Female
				Rie staging
2	0	0	0	0
0	0	6	6	1
8	4	6	0	2
8	0	6	0	3
118.80	35.00	211.33	95.00	WBC (¬10^3^/mm3)
107.00	110.00	105.00	130.00	Hb (g/dl)
10.62	13.00	10.60	11.00	Plt (10^3^/mm3)
50.00	00.00	0.00	33.33	Splenomegaly (%)
10.00	00.00	11.11	100.00	Axillary lymphadenopathy (%)
00.00	00.00	0.00	0.00	Ventral lymphadenopathy (%)
20.00	50.00	22.22	0.00	Cervical lymphadenopathy (%)

In an effort to evaluate clinical signs, Rie staging was determined for patients, but due to limited information on sites of lymphadenopathies, Binet staging was not defined. Clinical and laboratory findings of whole patients are described in Table 3.

**Table 3:** Clinical findings of 22 CLL patients

**sex**	**age**	**Genomic** **alteration**	**splenomegaly**	**lymphadenopathy**	**WBC×10** ^3^	**Plt×10** ^3^	**Hb**
M	60	Del13q14	Pos	Cervical	89	210	**12**
M	59	NO	Neg	Axillary	65	190	**10**
F	65	Del13q14 Del17p	Neg	Axillary	95	130	**11**
M	60	NO	Pos	Axillary & cervical	110	150	**8**
M	45	NO	Neg	Neg	150	230	**12**
M	50	NO	Pos	Cervical	150.2	110	**12.5**
M	71	Del13q14	Neg	Axillary	450	80	**9**
M	56	Del11q	Neg	Axillary	35	135	**13**
M	55	NO	Pos	Axillary & ventral	130	220	**11**
M	53	Del13q14	Neg	Axillary	161	175	**11**
F	67	Del13q14	Pos	Axillary	157.5	179	**9.5**
M	62	Del13q14	Pos	Axillary	85	190	**11**
M	55	NO	Neg	Axillary	69.2	183	**10.5**
F	61	Del17q	Neg	Axillary	92	137	**9.5**
M	57	No	Pos	Axillary &ventral	113	170	**10**
M	50	No	Neg	No	470	215	**10.5**
M	53	No	Pos	Axillary	142	100	**12**
M	53	Del13q	Neg	Axillary	138	75	**10.5**
M	53	Del11q	Neg	Axillary& cervical	100	155	**12**
M	58		Pos	Axillary & ventral	65	230	**12.5**
M	59	Del13q	Neg	Axillary &ventral	159.4	182.5	**11.7**
F	63	Del13q	Neg	Axillary	159	191	**8.8**

## Discussion

 Cytogenetic methods are recognized as practical methods to detect chromosomal abnormalities. There are two reasons for using I- FISH for B- CLL diagnosis; first, long deletion sites in chromosomes makes it impossible to design a primer and to perform RT - PCR; second, B cells did not stimulate as well as T Cells in culture, and a proper karyotype will not be gained. Because of these restrictions, I FISH is a practical technique to detect chromosomal disorders in B-CLL malignancies.

As mentioned earlier, CLL chromosomal aberrations can be detected in more than 80 % cases^4^^,^^9^^. ^Some aberrations can affect clinical course and disease outcome. It's believed that chromosomal abnormalities can be available as prognostic markers^6^^, ^^9^^, ^^13^^, ^^14^^,^^16^. 

Gender ratio is in favor of male, since, many B-CLL patients are men in Teimori et al’s study and women were unwilling to take part in their study is the reason^4^. Similarly, we had 19 men and 5 women in the present study (ratio 3.8).

Del 13q34 and 13q14 are known as the most common types of abnormalities in studied population of Berkova's research. Our results is similar to the mentioned findings^5^. In two other studies conducted by Calin et al (2002) and Klein et al (2010) deletion of 13q14 region associated with favorable prognosis was detected in relatively 50% of CLL patients. MiR-15a and miR16-1 are located in this deleted region and have been described to affect tumor suppressor activity of CLL^21^^,^^22^^.^ Alongside these microRNAs, other genes such as DLEU7 are located in 13q region. It can impress tumor suppressor activity and different clinical courses of disease. Overall, 13q deletions induce biological heterogeneity of CLL cases; it has been proved by gene expression profiling and miRNA analysis^23^^,^^24^. In the current study, like other surveys we detected del13q14 in 40-90% of patients. Based on the obtained results, WBC count was high among del13q14 patients (P>0.05), anemia was more obvious among del13q patients and del13q14 was higher in women (60% vs. 31.56%).

Contrary to Hartmut Dohner's findings, none of the patients with del13q14 had splenomegaly (it was observed in more than 50 % of Hartmut's study participations with mutation noted above) the lymphadenopathy occurred mostly in cervical lymph nodes, and most of lymphadenopathies were observed in abdomen site^10^. Meanwhile, normal karyotype frequency was 41.66% among our studied population, while it was 18% in Hartmutet al.’s study ^6^. 

Deletion of the long arm of chromosome 11 is seen in 5-20% of CLL patients^6^^,^^25^^,^^26^.The minimal deleted region including 11q22 ATM gene has been studied frequently in cases of CLL with del(11q), but it has been detected in 8-30% of 11q-patients^27^.This finding shows that other genes have important role in 11q deletions. One of these genes is BIRC3, which is located near ATM gene^28^. However, the study by Rose-Zerilliet et al. indicates that ATM mutations compared to BIRC3 deletion, are more effective on progression-free survival and overall survival in 11q-deleted patients who are treated with first–line therapy^29^. CLL patients with del(11q) are identified by large and multiple lymphadenopathies and have poor prognostic factors such as unmutated IGHV genes. Although Patients with del11q have generally accelerated disease progression, chemo immunotherapy treatment may overcome the adverse prognostic impacts of del11q on untreated patients^30^.Moreover, 9.09% of our study participants with del11q and the lowest WBC count had cervical lymphadenopathy.

As it has been discovered, ATM is affected by Del 11q22-q23^6^. Frequency of this anomaly is nearly 10-20%, which is similar to the results achieved by our study (13.63%).

Deletion of 17p is observed in 3-8% of CLL patients in Delgao’s study^31^.In another study, it was detected in 30% of patients^32^^-^^33^.Deletion of short arm of chromosome 17 is one of the most common acquired abnormalities which is frequent after treatment, and can also be seen in other hematologic abnormalities such as diffuse large B-cell lymphoma, non-Hodgkin^'^s lymphomas or mantle cell lymphoma^5^. CLL patients with 17p deletion are classified into the highest-risk group having the shortest overall survival. According to the clinical point of view, new studies show clinical heterogeneity in 17p CLL patients based on manifestations of this abnormality as an early event (de novo) ,or most frequently, as a secondary change^34^. Patients with an early del17p have shown longer overall survival, but those with acquired 17p have significant decreased survival^35^. Patients with del17p have unusual immunophenotype with strong CD20, CD79b, FMC7, surfaceIg, expression of CD38, ZAP70, and unmutated IGHV. The above-mentioned factors suggest poor prognosis in cases del17p^36^^-^^38^^.^ Similarly, in our study, patients with del17p have also atypical immunophenotype with higher intensity of CD20 and CD19.

Baliakas et al. evaluated 1001 European CLL patients; of who 65% had aberrant karyotype. Chromosomal translocations such as 13q, 14q, 18q, 17p, 5q and 11q were assessed. They were reported in 32% of studied individuals; of whom 71.87% had just one aberration and 22.08%, 4.37% and 1.56% had 2, 3 and≥ 4 translocations, respectively. Generally, 15.7% of Baliakas's study and 2.08% of participants in the present study had that one abberration^39^. It seems that this difference is due to frequency of studied population.

One study suggests that allogeneic stem cell transplantation is the best strategy for patients with del17p, who are not in complete remission^40^. Other studies have reported that patients with anomaly of short armof chromosom17 will inactive a tumor suppressor protein, p53, which affects disease prognosis. It has been reported in 5.2% of cases studied by Berkova in Czech in 2009^15^, and 9.09%of cases in this study. These mentioned mutations are not rare and result in disease progression^41^^,^^42^.

Statistical analysis of our patients’ data revealed a significant correlation between age and splenomegaly (P value<0.05). According to the results of this study, splenomegaly is less frequent in younger patients. There was a significant correlation between chromosomal changes and age in such patients (p value< 0.05).

## CONCLUSION

 I-FISH is known as the gold standard manner to assess genomic changes in B-CLL, and is more time- saving in laboratory usage, especially in clinical diagnosis procedures. As mentioned earlier, 13q14 deletion was the most common abnormality and normal karyotype was the less frequent type among our studied populations. It is suggested to perform similar study on B-CLL cases in a larger population to discover between patients' age and chromosomal aberrations. One of the limitations of this study was IGH mutation which is necessary to apply as a discriminative tool for CLL and mantel cell lymphoma.

## References

[1] Coll-Mulet L, Gil J (2009). Genetic alterations in chronic lymphocytic leukaemia. Clin Transl Oncol.

[2] Šindelářová L, Michalová K, Zemanová Z (2005). Incidence of chromosomal anomalies detected with FISH and their clinical correlations in B-chronic lymphocytic leukemia. Cancer genet and cytogenet.

[3] Wren C, Moriarty H, Marsden K (2010). Cytogenetic investigations of chronic lymphocytic leukemia. Cancer genet and cytogenet.

[4] Teimori H, Ashoori S, Akbari MT (2013). FISH Analysis for del6q21 and del17p13 in B-cell Chronic Lymphocytic Leukemia in Iranians. Iran Red Crescent Med J.

[5] Puiggros A, Blanco G, Espinet B (2014). Genetic Abnormalities in Chronic LymphocyticLeukemia: where we are and where we go. Biomed Res Int.

[6] Döhner H, Stilgenbauer S, Benner A (2000). Genomic aberrations and survival in chronic lymphocytic leukemia. N Engl J Med.

[7] Binet J, Leporrier M, Dighiero G (1977). A clinical staging system for chronic lymphocytic leukemia. Prognostic significance. Cancer.

[8] Rai KR, Sawitsky A, Cronkite EP (1975). Clinical staging of chronic lymphocytic leukemia. Blood.

[9] Dicker F, Schnittger S, Haferlach T (2006). Immunostimulatory oligonucleotide-induced metaphase cytogenetics detect chromosomal aberrations in 80% of CLL patients: a study of 132 CLL cases with correlation to FISH, IgVH status, and CD38 expression. Blood.

[10] Moreno C, Montserrat E (2008). New prognostic markers in chronic lymphocytic leukemia. Blood Rev.

[11] Rai KR, Döhner H, Keating MJ Chronic lymphocytic leukemia: case-based session. Hematology Am Soc Hematol Educ Program.

[12] Stevens-Kroef MJ, van den Berg E, Olde Weghuis D (2014). Identification of prognostic relevant chromosomal abnormalities in chronic lymphocytic leukemia using microarray-based genomic profiling. Mol cytogenet.

[13] Rigolin GM, Cibien F, Martinelli S (2012). Chromosome aberrations detected by conventional karyotyping using novel mitogens in chronic lymphocytic leukemia with “normal” FISH: correlations with clinicobiologic parameters. Blood.

[14] Stilgenbauer S, Bullinger L, Benner A (1999). Incidence and clinical significance of 6qdeletions in B cell chronic lymphocytic leukemia. Leukemia.

[15] Berkova A, Zemanova Z, Trneny M (2009). Clonal evolution in chronic lymphocytic leukemia studied by interphase fluorescence in-situ hybridization. Neoplasma.

[16] Reddy K (2006). Chronic lymphocytic leukaemia profiled for prognosis using a fluorescence in situ hybridisation panel. Br J Haematol.

[17] Turgut B, Vural O, Pala FS (2007). 17p Deletion is associated with resistance of B-cell chronic lymphocytic leukemia cells to in vitro fludarabine-induced apoptosis. Leuk Lymphoma.

[18] Baliakas P, Iskas M, Gardiner A (2014). Chromosomal translocations and karyotypecomplexity in chronic lymphocytic leukemia: a systematic reappraisal of classic cytogenetic data. Am J Hematol.

[19] Stilgenbauer S BL, Lichter P, Döhner H, the German CLL Study Group (GCLLSG) (2002). Genetics of chronic lymphocytic leukemia: genomic aberrations and VH gene mutation status in pathogenesis and clinical course. leukemia.

[20] Smoley SA, Van Dyke DL, Kay NE (2010). Standardization of fluorescence in situ hybridization studies on chronic lymphocytic leukemia (CLL) blood and marrow cells by the CLL Research Consortium. Cancer Genet Cytogenet.

[21] Calin GA, Dumitru CD, Shimizu M (2002). Frequent deletions and down-regulation ofmicro-RNA genes miR15 and miR16 at 13q14 in chronic lymphocytic leukemia. Proc Natl Acad Sci U S A.

[22] Klein U, Lia M, Crespo M (2010). The DLEU2/miR-15a/16-1 cluster controls B cell proliferation and its deletion leads to chronic lymphocytic leukemia. Cancer cell.

[23] Mosca L, Fabris S, Lionetti M (2010). Integrative genomics analyses reveal molecularlydistinct subgroups of B-cell chronic lymphocytic leukemia patients with 13q14 deletion. Clin Cancer Res.

[24] Rodrguez AE, Hernndez J, Benito R (2012). Molecular characterization of chronic lymphocytic leukemia patients with a high number of losses in 13q14. PLoS One.

[25] Marasca R, Maffei R, Martinelli S (2013). Clinical heterogeneity of de novo 11q deletion chronic lymphocytic leukaemia: prognostic relevance of extent of 11q deleted nuclei inside leukemic clone. Hematol Oncol.

[26] Zenz T, Mertens D, Kuppers R (2010). From pathogenesis to treatment of chronic lymphocytic leukaemia. Nat Rev Cancer.

[27] Ouillette P, Li J, Shaknovich R (2012). Incidence and clinical implications of ATM aberrations in chronic lymphocytic leukemia. Genes, Chromosomes and Cancer.

[28] Rossi D, Fangazio M, Rasi S (2012). Disruption of BIRC3 associates with fludarabine chemo refractoriness in TP53 wild-type chronic lymphocytic leukemia. Blood.

[29] Rose-Zerilli MJ, Forster J, Parker H (2014 ). ATM mutation rather than BIRC3 deletion and/or mutation predicts reduced survival in 11q-deleted chronic lymphocytic leukemia: data from the UK LRF CLL4 trial. Haematologica.

[30] Tsimberidou AM, Tam C, Abruzzo LV (2009). Chemoimmunotherapy may overcome the adverse prognostic significance of 11q deletion in previously untreated patients with chronic lymphocytic leukemia. Cancer.

[31] Delgado J, Espinet B, Oliveira AC (2012). Chronic lymphocytic leukaemia with 17p deletion: a retrospective analysis of prognostic factors and therapy results. Br J Haematol.

[32] Stilgenbauer S, Zenz T, Winkler D (2009). Subcutaneous alemtuzumab in fludarabine-refractory chronic lymphocytic leukemia: clinical results and prognostic marker analyses fromthe CLL2H study of the German Chronic Lymphocytic Leukemia Study Group. J Clin Oncol.

[33] Wawrzyniak E, Kotkowska A, Blonski JZ (2014). Clonal evolution in CLL patients as detected by FISH versus chromosome banding analysis, and its clinical significance. Eur J Haematol.

[34] Landau DA, Carter SL, Stojanov P (2013). Evolution and impact of subclonal mutations in chronic lymphocytic leukemia. Cell.

[35] Tam CS, Shanafelt TD, Wierda WG (2009). De novo deletion 17p13. 1 chronic lymphocytic leukemia shows significant clinical heterogeneity: the MD Anderson and Mayo Clinic experience. Blood.

[36] Quijano S, Lopez A, Rasillo A (2008). Impact of trisomy 12, del (13q), del (17p), and del (11q) on the immunophenotype, DNA ploidy status, and proliferative rate of leukemic B‐cells in chronic lymphocytic leukemia. Cytometry B Clin Cytom.

[37] A Krober JB, S HafnerHafner S, Bühler A (2006). Additional genetic high-risk features such as 11q deletion, 17p deletion, and V3- 21 usage characterize discordance of ZAP-70 and VH mutation status in chronic lymphocytic leukemia. J Clin Oncol.

[38] Rassenti LZ, Jain S, Keating MJ (2008). Relative value of ZAP-70, CD38, and immunoglobulin mutation status in predicting aggressive disease in chronic lymphocytic leukemia. Blood.

[39] Baliakas P1, Iskas M, Gardiner A (2014). Chromosomal translocations and karyotype complexity in chronic lymphocytic leukemia: a systematic reappraisal of classic cytogenetic data. Am J Hematol.

[40] Jain N, O'Brien S (2012). Chronic lymphocytic leukemia with deletion 17p: emerging treatment options. Oncology (Williston Park).

[41] Gonzalez D, Martinez P, Wade R (2011). Mutational status of the TP53 gene as a predictor of response and survival in patients with chronic lymphocytic leukemia: results from the LRFCLL4 trial. J Clin Oncol.

[42] Gribben JG Molecular profiling in CLL. Hematology Am Soc Hematol Educ Program.

